# Love Components in Free-Choice and Arranged Marriages Among Five Non-Western Populations From Africa, Amazonia, and Himalayas

**DOI:** 10.1007/s10508-024-03040-y

**Published:** 2024-12-13

**Authors:** Piotr Sorokowski, Agata Groyecka-Bernard, Marta Kowal, Marina Butovskaya, Michal Mikolaj Stefanczyk, Tomas Huanca, Amit Kumar, Upma Manral, Oneyekachi M. Odo, Ike E. Onyishi, Wiktoria Jędryczka

**Affiliations:** 1 Institute of Psychology, University of Wrocław, ul. Dawida 1, 50-527 Wrocław, Poland; 2 Being Human Lab, Institute of Psychology, University of Wrocław, Wrocław, Poland; 3 Institute of Ethnology and Anthropology, Russian Academy of Sciences, Moscow, Russia; 4 Russian State University for Humanities, Moscow, Russia; 5 Centro Boliviano de Investigación y Desarrollo Socio Integral, San Borja, Bolivia; 6 Wildlife Institute of India, Dehradun, India; 7 Department of Psychology, University of Nigeria, Nsukka, Nigeria

**Keywords:** Love, Free-choice marriages, Arranged marriages, Cross-cultural

## Abstract

Two main ways to enter a marriage are through free choice and through an arrangement between families, known as an arranged marriage. In this study, we compared differences in three dimensions of love (Intimacy, Passion, and Commitment) between spouses in love-based marriages and arranged marriages among five non-Western societies: Bhotiya from the Himalayas, Igbo from Nigeria, Kimeru from Kenya, Meru from Tanzania, and Tsimane’ from Bolivia. When considering all data gathered from the five cultures, free choice and arranged marriages did not differ significantly in average love scores. However, within cultures, some differences did emerge between free choice and arranged marriages, such as spouses from free choice marriages from Bhotiya and Tsimane’ culture showing higher levels of Intimacy than those from arranged marriages. However, in the Bhotiya, this difference was only evident in marriages lasting longer than 10 years. Moreover, spouses from free choice marriages from Bhotiya reported higher levels of passion, while spouses from free choice marriages from Tsimane’ reported higher levels of commitment than individuals from arranged marriages. Conversely, wives from arranged marriages from Meru culture from Tanzania reported higher levels of Intimacy and Passion as compared to wives from free choice marriages. In Kimeru and Igbo, no significant differences were found. These results contribute to the growing body of literature on the relationship between culture, love, and marriage. Our data also challenge the Western perception that arranged marriages lack love.

**Supplementary Information:**

The online version contains supplementary material available at 10.1007/s10508-024-03040-y.

## Introduction

Forms of committed relationships between men and women are ubiquitous across cultures (Bell, 1997; Myers et al., 2005). One of such relationships is marriage, typically defined as a legally and socially recognized union, entailing significant economic, social, and sexual obligations for both partners and their families (Bachrach et al., 2000). Although marriage is one of the most fundamental human relationships worldwide (Larson & Holman, 1994), the specific customs surrounding entering such a union vary both between and within cultures (Goode, 1963; Hamon & Ingoldsby, 2003; Levy Jr., 2013; Rabin & Rahav, 1995). Marriages can be broadly categorized as either marriages of choice (also called free choice marriages*)*, resulting from the mutual decision of two partners or marriages arranged by the families of the future spouses (Agey et al., 2021).

There is a widespread belief that romantic relationships resulting from the choice of the future spouses are filled with more love than those arranged by families (De Munck, 1998). However, given that most of the studies on love, marriage, and mating strategies are predominantly conducted in Western contexts (but see e.g.: Conroy-Beam et al., 2019; Kowal et al., 2024; Sorokowski et al., 2023; Walter et al., 2020), the assumption regarding the superiority of free choice marriages may be flawed. Our research was designed and conducted to address this gap.

The debate over whether one type of marriage (arranged or free choice) results in greater marital satisfaction or love, although longstanding (Blood, 1967), remains inconclusive and sparsely addressed. Some studies have reported more positive feelings in free choice than in arranged marriages in various cultural contexts, such as in China (Pimentel, 2000), Israel (Lev-Wiesel & Al-Krenawi, 1999), Nepal (Allendorf & Ghimire, 2013), Turkey (Hortaçsu & Oral, 1994), or India (Myers et al., 2005). On the other hand, some studies suggested that individuals in arranged marriages, compared to free choice, exhibit more positive attitudes toward their partners, as evidenced in Japan (Blood, 1967) or India (Madathil & Benshoff, 2008). Conversely, other research has found no significant differences between the two types of marriages, as observed in India (Mir et al., 2016), Israel (Shachar, 1991), and Nepal (Hoelter et al., 2004).

Another interesting aspect is the love dynamic within a relationship over time. An old saying suggests that “love matches start out hot and grow cold, while arranged marriages start out cold and grow hot” (as cited in Xiaohe & Whyte, 1990), implying that arranged marriages might be more advantageous in the long run than free choice marriages. Initial evidence supporting this notion was provided by Blood (1967), who observed increasing marital quality over time in arranged marriages compared to free choice marriages in Japan. Nevertheless, subsequent research has contradicted these findings. Xiaohe and Whyte (1990) replicated Blood’s (1967) study with Chinese couples and observed fluctuating patterns of marital quality, with those in free choice marriages reporting higher closeness and lower discord compared to spouses from arranged marriages. Additionally, couples who entered relationships by choice were less likely to divorce.

In our study, we relied on Sternberg’s (1986) triangular theory of love, which posits that love consists of three components: intimacy, passion, and commitment. Intimacy refers to the trust and friendship shared with a partner, passion relates to intense feelings and desire towards the loved one, and commitment refers to a conscious decision to maintain the relationship. Based on the intensity of experiencing each of these components, Sternberg distinguished eight profiles of love. For instance, romantic love entails high intimacy and passion but low commitment. Empty love, on the other hand, is characterized by high commitment but low intimacy and passion. Given that the intensity of these components fluctuates over time, the experienced type of love may transition to other forms, such as consummate love, with all three components high, or companionate love, with high intimacy and commitment but low passion. Importantly for this project, the components of Sternberg’s triangular theory of love can be meaningfully measured across cultural contexts (Kowal, 2023; Kowal et al., 2024; Sorokowski et al., 2023).

In the present work, we utilized the opportunity afforded by our international collaboration and analyzed the facets of love experienced by individuals from two types of marriages (free choice and arranged marriages) across five non-Western and less industrialized societies, including Igbo from Nigeria, Bhotiya from Himalayas, Meru from Tanzania, Kimeru from Kenya, and Tsimane’ from Bolivia. Below, we provide a brief description of each of the studied societies (for an overview, see Table S1 in the Supplementary Material).

### West Africa: Igbo (Nigeria)

The Igbo people, an African ethnic group, inhabit Igboland in the south-central and south-eastern parts of Nigeria. With a population of about 34 million, mainly concentrated in Nigeria, they maintain a traditional agrarian lifestyle. Our study was conducted in a rural Igbo setting in Enugu state, located in the south-eastern region of Nigeria. The Igbo have been intensively studied by sociologists, anthropologists, and psychologists (e.g., Okeke et al., 2017; Okemgbo et al., 2002; Onyishi et al., 2012). Marital practices among the Igbo can be either arranged or based on the consent of the partners. In the past, when economic factors held more significance, polygamy, reflecting a multi-generational family model suited to agrarian economies was prevalent. However, due to European-colonial influences and subsequent cultural changes (Abidogun, 2007), monogamy and nuclear family structures have become more widespread.

### South Asia: Bhotiya (Himalayas)

The Bhotiya people live in the Himalayas and are ethnolinguistically related to the Tibetan people. Our study was conducted among the Tolchha and Marchha Bhotiya subgroups, indigenous communities of Indo-Mongoloid origin residing in the Niti valley within the Nanda Devi Biosphere Reserve in Uttarakhand, India. Marriages among the Bhotiya are typically arranged by parents (Bergmann et al., 2008), although free choice marriages also occur. For more information about this group, see works of Mitra et al. (2013) and Bergmann et al. (2008).

### East Africa: Meru (Tanzania)

The Meru people (Wameru, Rwa, Warvo) constitute an ethno-linguistic group with 26 clans residing in Tanzania (Butovskaya et al., 2016). They adhere to a traditional agrarian lifestyle, with a population numbering around 2 million. Our study was conducted among Meru living around Arusha and Mount Meru, a volcano in north-east of Tanzania. The Meru society has been extensively studied in historical, cultural, and social context (see Butovskaya et al., 2016; Ceppi & Nielsen, 2014). Presently, the Meru practice both arranged and non-arranged monogamy. Noteworthy, the Meru people share the same name with the Meru people of Kenya, but they are distinct ethnic groups with their own unique histories and identities (Fadiman, 1973; Puritt, 1970).

### East Africa: Kimeru (Kenya)

The Kimeru (Meru) are the indigenous inhabitants of central Kenya, with a population of approximately 1.3 million people (Appiah & Gates, 2010). The sample in the present study were the Kimeru living near the small market towns of Laare and Mutuati, situated 300 km northeast of Nairobi, Kenya’s capital. The Kimeru society has been extensively studied by historians, ethnographers, and sociologists (see Jaetzold et al., 2006; Marczak & Sorokowski, 2018). The Kimeru adhere to a patriarchal social structure, and marriages are often arranged by the woman’s parents and the man seeking to marry their daughter (Nyaga, 1997). While monogamy is prevalent among the Kimeru today, polygamy is still occasionally practiced (Wamue & Getui, 1996), especially among wealthy and high-status individuals men (Mengisteab & Hagg, 2017; Nyaga, 1997).

### South America: Tsimane’ (Bolivia)

The Tsimane’ are a native Amazonian society of farmer-foragers, with a population of about 8,000, spreading across approximately 100 villages, predominantly in the Beni area of northern Bolivia. Our study was conducted in several villages within the Maniqui river region, including Campo Bello, Las Palmas, Las Minas, Uachichi, Maracas, Catumare, and Anachere. The Tsimane’ have been extensively documented in the literature (e.g., Huanca, 2008; Ringhofer, 2010). Similar to other indigenous Amazonian societies, the Tsimane’ still practice cross-cousin marriage (i.e., marrying the child of one’s parent’s sibling). Traditionally, marriage among Tsimane’ is arranged by parents (Winking et al., 2011), although recent evidence suggests that many couples claim to have formed without parental influence (Sorokowski et al., 2017a).

To take advantage of data coming from such diverse samples, our study examined differences in three subscales of love (Intimacy, Passion, and Commitment), as measured by the Triangular Love Scale (Sternberg, 1997) between individuals from free choice and arranged marriages. We hypothesized that free choice marriages might exhibit higher levels of intimacy and passion, while arranged marriages might demonstrate higher levels of commitment. Furthermore, we expected differences between free choice and arranged marriages in the trajectory of love feelings over time, with free choice marriages presenting higher levels of love irrespective of marriage duration and arranged marriages presenting growing levels of love across time. We tested these predictions in a series of moderated analyses, regressing love components (Intimacy, Passion, and Commitment) on the marriage type, marriage duration, and their interaction in each of the societies.

## Method

The study was conducted in 2012–2020. Each time, one or more team members visited the given location and conducted field studies with the assistance of local translators and guides. All the participants gave their informed consent to be included in the research.

### Participants

**Fig. 1:**
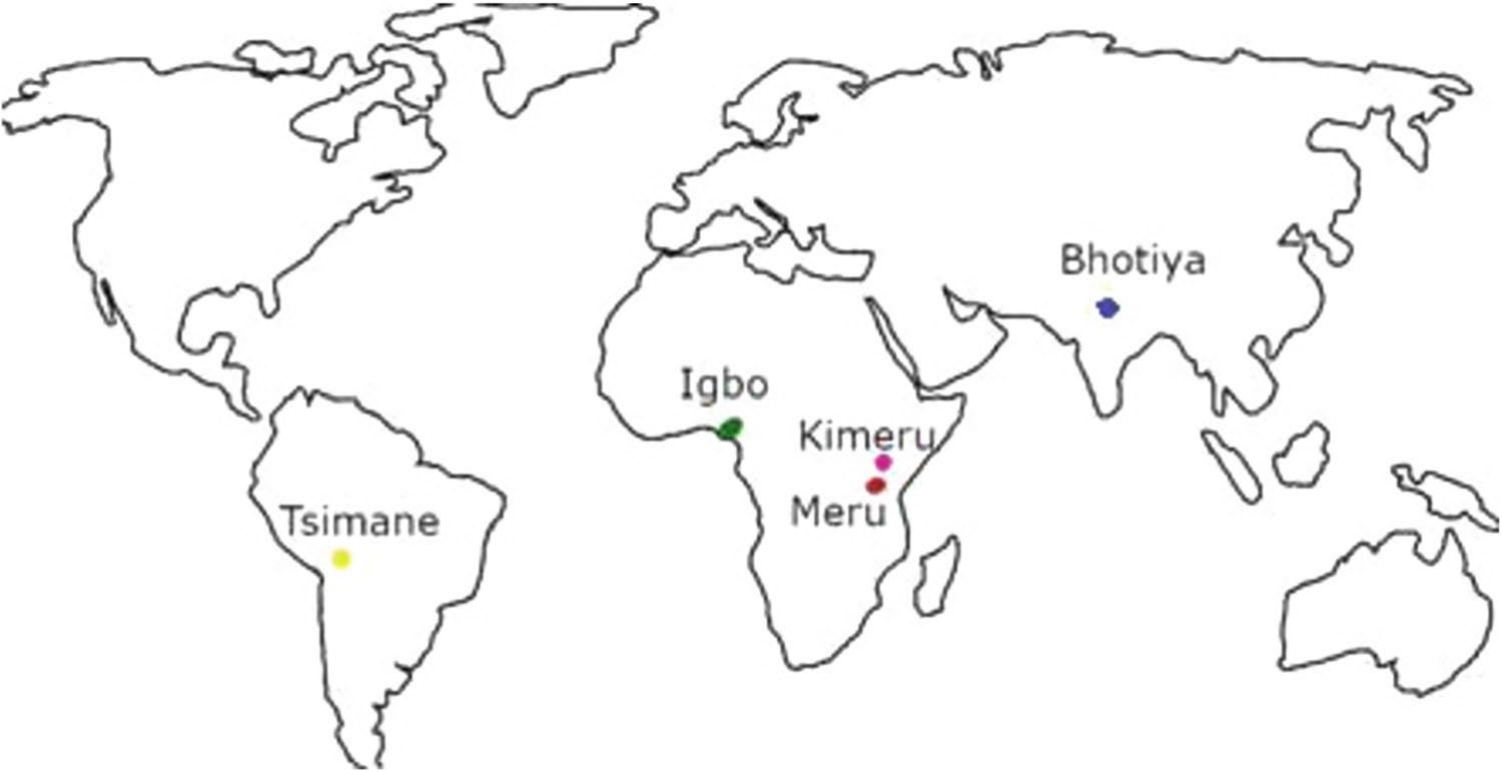
Geographical location of the studied societies

Five prominently different ethnic groups were included in the study, all of which are from geographically and culturally distinct parts of the world (see Fig. 1). Those were: Bhotiya (*n* = 110, women = 55), Igbo (*n* = 98, women = 47), Kimeru (*n* = 124, women = 65), Meru (*n* = 118, all women), and Tsimane’ (*n* = 148, women = 75). Despite minor differences between locations, the recruitment of subjects followed a very similar process. The team members, which always included a local researcher familiar with the area and population, first obtained consent from local leaders to conduct the research. They then announced in location X or Y (see above) that the project would take place. All individuals eligible for the study (e.g., married persons) were invited to participate. Only willing participants took part in the study. Each participant was compensated for their involvement, with the form of compensation varying by location—ranging from a small sum of money (equivalent to USD 3–10) to small gifts, such as fish hooks and flour. In each population, we included data only from participants who reported either making the decision to marry their partner independently (free choice marriage) or having the marriage arranged by a third party.

### Measures

To assess the type of marriage, participants were asked, “Did you choose with whom you would marry, or was your spouse chosen for you, by, for instance, your family?” Responses were coded as arranged marriages (0) or free choice marriages (1). If participants were unsure, further love-related questions were not collected. Additionally, participants were asked to indicate the duration of their marriages in years.

Since participants were not accustomed to completing surveys, we employed a shortened and simplified version of Sternberg’s (1986) Triangular Love Scale, previously utilized in studies on the Tanzanian Hadza (Sorokowski et al., 2017b). This simplified version consists of 16 items; five items measuring Intimacy (e.g., “I share deeply personal information about myself with my partner”; McDonald’s ω = 0.63), six items measuring Passion (e.g., “I find myself thinking about my partner frequently during the day”; McDonald’s ω = 0.75), and five items measuring Commitment (e.g., ‘I view my relationship with my partner as permanent’; McDonald’s ω = 0.63; see the Supplementary Material for all items). The questionnaire was translated into the languages spoken by the respective societies with the assistance of local translators. Data were coded such that higher numbers indicate higher levels of love (*1* – no love and *3* – certain love). Participants responded verbally to the questions read aloud to them.

## Results

The analyses were performed in r (Version 4.2.0.). Descriptive statistics are presented in Table 1. As a first step, we compared the levels of the three love components across societies using one-way ANOVA. Due to heterogeneity of variance, we report Welch’s *F* statistic. Our analysis revealed a significant effect of culture on Intimacy, Passion, and Commitment (*F*(4, 272.08) = 30.27, *p* < 0.001, *F*(4, 285.29) = 29.44, *p* < 0.001, *F*(3, 265.66) = 6.72, *p* < 0.001, respectively). Bhotiya exhibited the highest scores for all three love components, followed by Igbo, Kimeru, Tanzanian Meru, and Tsimane’. Mean levels of love across societies and detailed Games-Howell post-hoc analyses are available in the Supplementary Material. When analyzing all data together, the multilevel models with the three love components regressed on the marriage type, marriage duration, and their interaction, with participants nested within cultures, showed no significant relationships (see Table 2). However, considering the differences in reported love levels across cultures, each culture was treated independently in the subsequent analyses.

**Table 1:** Frequencies of marriage type and descriptive statistics

Marriage type	Bhotiya				Igbo				Kimeru				Meru (Tanzania)				Tsimane’			
Mean age (in years)	31.60 (5.9)				33.00 (6.69)				40.60 (11.80)				27.20 (5.11)				33.80 (14.00)			
	N	I*	P*	C*	N	I*	P*	C*	N	I*	P*	C*	N	I*	P*	C*	N	I*	P*	C*
Free-choice	42	2.99 (0.04)	2.94 (0.12)	3 (0)	15	2.81 (0.31)	2.68 (0.44)	2.84 (0.24)	107	2.86 (0.26)	2.79 (0.34)	2.78 (0.33)	21	2.52 (0.48)	2.29 (0.59)	2.60 (0.52)	48	2.71 (0.40)	2.56 (0.37)	2.80 (0.32)
Arranged	68	2.94 (0.13)	2.83 (0.24)	3 (0)	83	2.87 (0.27)	2.74 (0.37)	2.89 (0.18)	17	2.74 (0.32)	2.88 (0.26)	2.84 (0.24)	97	2.75 (0.31)	2.56 (0.41)	2.77 (0.29)	100	2.51 (0.49)	2.46 (0.43)	2.70 (0.41)
Within culture comparisons of free choice and arranged marriages**																				
Intimacy	*t*(89.3) = 3.30, *p* = .001, *d* = .53				*t*(96) = -.70, *p* = .487, *d* = -.20				*t*(122) = 1.20, *p* = .233, *d* = .31				*t*(23.9) = -2.09, *p* = .047, *d* = -.66				*t*(48.8) = 2.52, *p* = .015, *d* = .58			
Passion	*t*(102.7) = 3.20, *p* = .002, *d* = .54				*t*(96) = -.59, *p* = .557, *d* = -.17				*t*(122) = -1.03, *p* = .305, *d* = -.27				*t*(24.3) = -2.02, *p* = .055, *d* = -.62				*t*(49.4) = 1.19, *p* = .241, *d* = .27			
Commitment					*t*(96) = -.89, *p* = .378, *d* = -.25				*t*(122) = -.66, *p* = .508, *d* = -.17				*t*(22.8) = -1.49, *p* = .151, *d* = -.51				*t*(42.4) = 2.01, *p* = .051, *d* = .50			

*Note*. *N* denotes the number of individuals, other numbers, if not otherwise specified, represent means, while standard deviations are presented in parentheses. *I: Intimacy P: Passion C: Commitment. ** Results of the comparison between free choice and arranged marriages within each culture: *t*-tests (with the unequal variances assumption for Bhotiya, Tanzanian Meru, and Tsimane’), and Cohen’s *d*s

**Table 2:** The results of the multilevel models with Intimacy, Passion, and Commitment regressed on marriage type, marriage duration, and their interaction. Participants were nested within cultures

Model	Fixed effect	Β	SE	95% CI	*p*
Intimacy					
	Marriage type^a^	− 0.060	0.048	[− 0.155, 0.035]	0.214
	Marriage duration	0.028	0.040	[− 0.051, 0.107]	0.482
	Marriage type*duration	− 0.064	0.040	[− 0.143, 0.015]	0.112
Passion					
	Marriage type^a^	0.019	0.048	[− 0.075, 0.113]	0.693
	Marriage duration	− 0.016	0.040	[− 0.094, 0.062]	0.693
	Marriage type*duration	0.005	0.040	[− 0.073, 0.083]	0.894
Commitment					
	Marriage type^a^	0.008	0.049	[− 0.087, 0.103]	0.868
	Marriage duration	0.027	0.040	[− 0.052, 0.107]	0.499
	Marriage type*duration	− 0.001	0.041	[− 0.081, 0.079]	0.981

*Note*. ^a^ Marriage type: Free choice marriage is the reference category; thus, a positive coefficient indicates that love was higher in arranged marriages, while a negative coefficient indicates that love was higher in free choice marriages

To test our predictions regarding the effect of marriage type, marriage duration (in years), and their interaction in each society, we conducted a series of moderated regressions. We utilized bootstrapping approach with 5,000 bootstrap samples to increase the robustness of the results. Bootstrapping is especially useful when analyzing smaller datasets (Dwivedi et al., 2017) consisting of scales with lower reliability (Miles et al., 1999) and skewed data (Russell & Dean, 2000). Each love component was treated as a dependent variable in a separate analysis, with marriage type serving as a moderator. All predictors were mean-centered and marriage type was contrast coded (− 1 for free choice and + 1 for arranged marriage). The results are presented in Table 3. In principle, if the link between marriage type and a given love component is significantly positive, it suggests that individuals in arranged marriages experienced higher love than those in free choice marriages. Conversely, a negative significant link indicates that individuals in free choice marriages experienced higher levels of love than those in arranged marriages. A positive significant association between a given love component and marriage duration implies that individuals in longer marriages experienced higher levels of love than those in shorter marriages, while a negative association suggests that individuals in longer marriages experienced lower levels of love than those in shorter marriages. If the interaction between marriage type and marriage duration is significant, it suggests that the relationship between love and marriage duration is moderated by marriage type. In other words, the trajectory of love over time differs between individuals in arranged marriages and those in free choice marriages.

**Table 3:** A summary of moderated regression models regressing Intimacy, Passion, and Commitment on marriage type, marriage duration, and their interaction

Love component	Parameter	Bhotiya (*n* = 110)		Igbo (*n* = 98)		Kimeru (*n* = 124)		Meru (*n* = 118)		Tsimane’ (*n* = 148)	
		B	95%CI	B	95%CI	B	95%CI	B	95%CI	B	95%CI
Intimacy	Marriage type^a^	** − 0.023**	**(− 0.039, − 0.011)**	0.020	(− 0.048, 0.158)	− 0.002	(− 0.108, 0.059)	**0.105**	**(0.030, 0.238)**	** − 0.129**	**(− 0.227, − 0.034)**
	Marriage duration	** − 0.003**	**(− 0.006, − 0.001)**	0.003	(− 0.014, 0.014)	− 0.003	(− 0.009, 0.002)	− 0.006	(− 0.047, 0.014)	**0.011**	**(0.003, 0.021)**
	Marriage type*duration	** − 0.004**	**(− 0.007, -0.002)**	− 0.003	(− 0.015, 0.014)	− 0.004	(− 0.010, 0.001)	− 0.002	(− 0.022, 0.039)	0.001	(− 0.007, 0.011)
Passion	Marriage type^a^	** − 0.039**	**(− 0.073, -0.010)**	0.037	(− 0.078, 0.233)	0.070	(− 0.020, 0.146)	**0.127**	**(0.016, 0.283)**	− 0.058	(− 0.149, 0.029)
	Marriage duration	** − 0.010**	**(− 0.015, − 0.004)**	− 0.001	(− 0.031, 0.013)	− 0.002	(− 0.011, 0.004)	− 0.012	(− 0.047, 0.010)	0.007	(− 0.002, 0.015)
	Marriage type*duration	− 0.004	(− 0.009, 0.002)	0.003	(− 0.010, 0.034)	− 0.002	(− 0.011, 0.003)	0.000	(− 0.023, 0.035)	0.006	(− 0.002, 0.015)
Commitment	Marriage type^a^			0.031	(− 0.044, 0.130)	0.059	(− 0.019, 0.119)	0.079	(− 0.004, 0.215)	** − 0.085**	**(− 0.181, -0.012)**
	Marriage duration			− 0.003	(− 0.019, 0.005)	− 0.001	(− 0.005, 0.003)	− 0.002	(− 0.046, 0.018)	0.001	(− 0.005, 0.009)
	Marriage type*duration			0.004	(− 0.003, 0.020)	− 0.004	(− 0.008, 0.000)	0.008	(− 0.013, 0.051)	0.001	(− 0.006, 0.008)

*Note.* ^a^Marriage type: Free choice marriage is the reference category; thus, a positive coefficient indicates that love was higher in arranged marriages, while a negative coefficient indicates that love was higher in free choice marriages. No model comparison was undertaken for predictors of commitment level in Bhotiya, because there was no variation in responses (and hence no variation to predict with regression). Bolded are significant results based on 95% CI

**Fig. 2:**
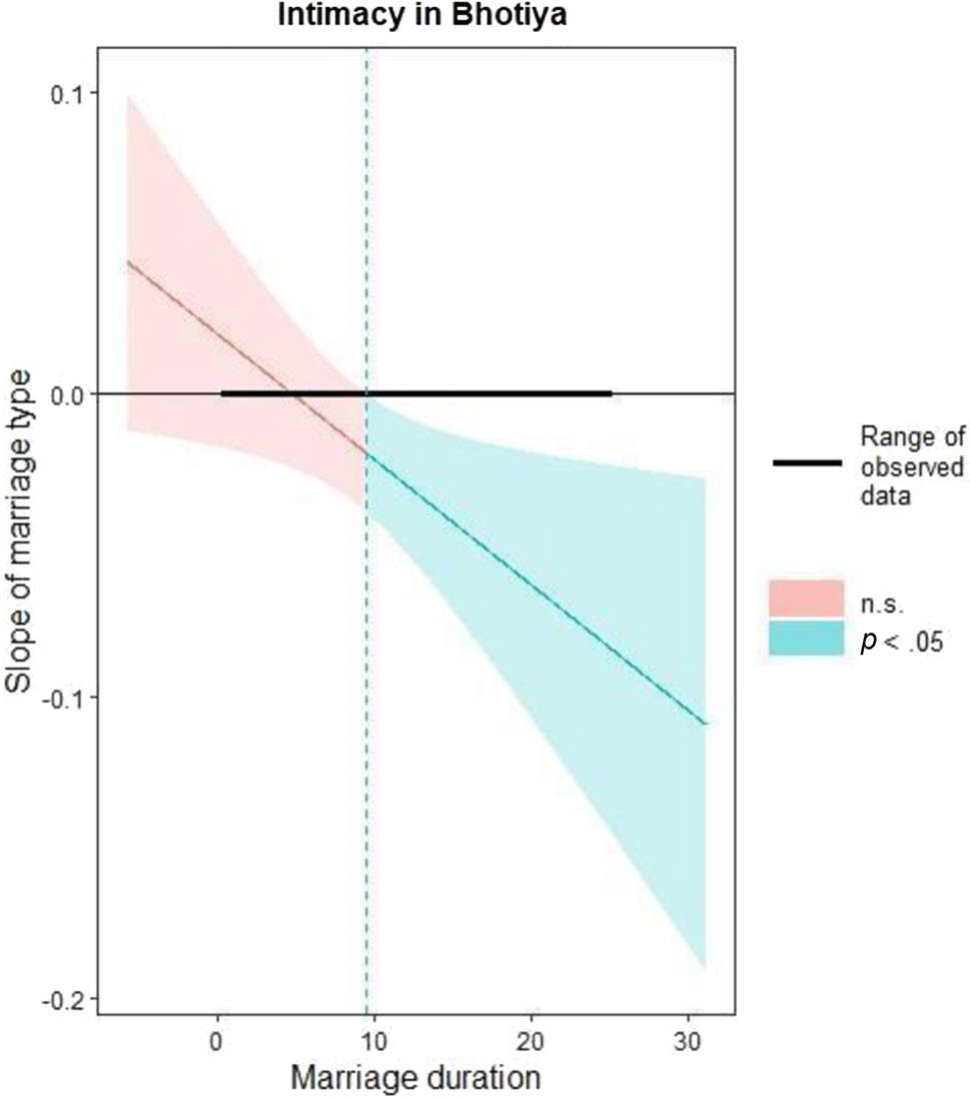
Johnson-Neyman regions of significance illustrating the interaction between marriage type (free choice and arranged marriages) and marriage duration in Bhotiya, with respect to Intimacy scores *Note.* Lower slope means that arranged marriages exhibited lower level of Intimacy compared to free choice marriages

**Fig. 3:**
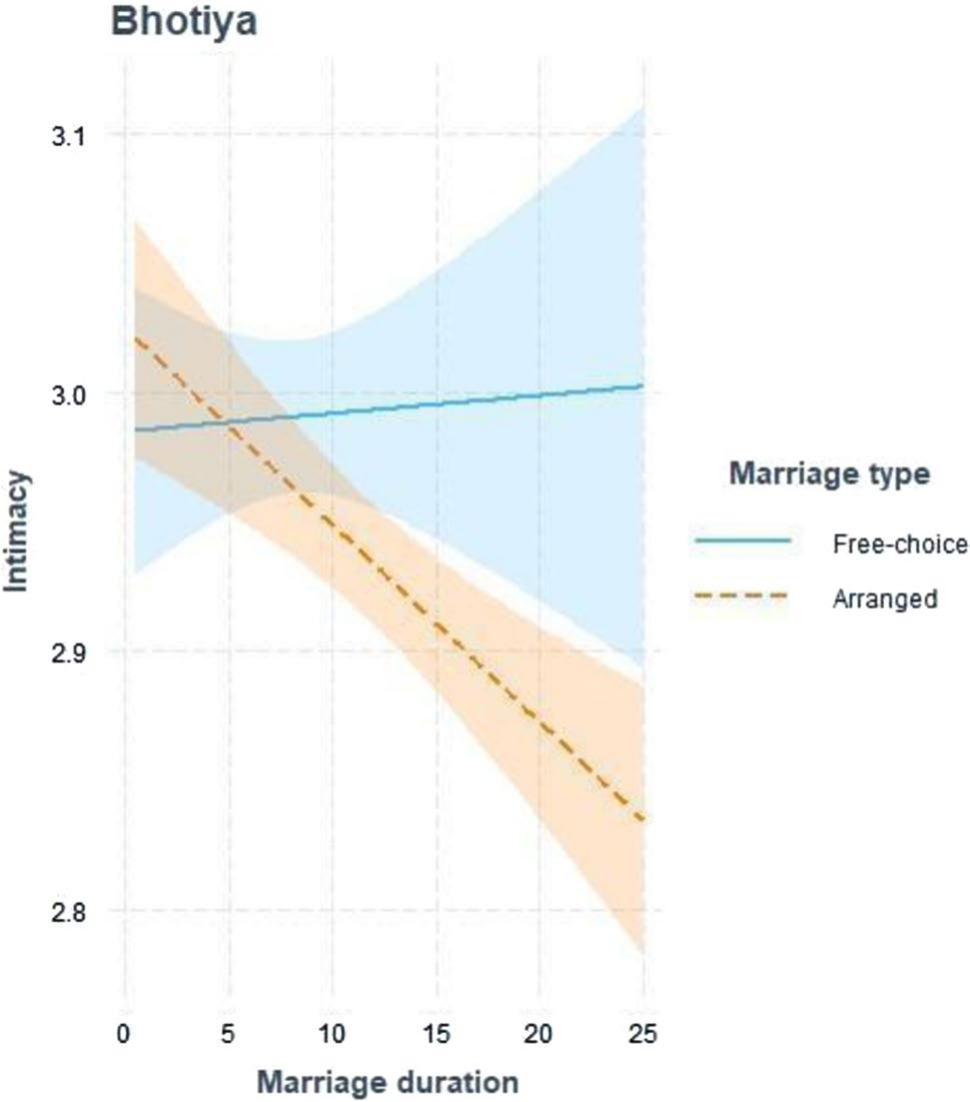
A graphical representation of the interaction between marriage type (free choice and arranged marriages) and marriage duration in Bhotiya, with respect to Intimacy scores. *Note.* Shaded area represents 95% confidence intervals

For Intimacy among Bhotiya, both predictors and their interaction were significant. Arranged marriages exhibited lower Intimacy scores compared to free choice marriages, and Intimacy showed a negative association with marriage duration. Further examination of the interaction with Johnson-Neyman regions of significance revealed that the relationship between marriage type and intimacy is insignificant for marriages lasting less than 10 years. However, beyond this threshold, the relationship became negative and significant, meaning that individuals from free choice marriages reported higher Intimacy over time than individuals from arranged marriages (see Fig. 2). A closer inspection of the interaction also revealed that the observed difference stemmed from lower Intimacy in arranged marriages that were longer in duration (see Fig. 3). Additionally, spouses from free choice marriages exhibited higher levels of Passion. Passion was also negatively related to marriage duration. We could not compute the analysis with Commitment as a dependent variable because of zero variance in this variable.

In the Tanzanian Meru, marriage type was related to Intimacy and Passion, with wives from arranged marriages reporting higher levels of these two love components than wives from free choice marriages. Conversely, among the Tsimane’, free choice marriages exhibited higher Intimacy and Commitment than arranged marriages. Furthermore, Intimacy was positively associated with marriage duration. We did not observe any significant relationships between love components and marriage type and marriage duration in the Igbo and Kimeru.

## Discussion

The main goal of our study was to investigate the commonalities and differences in levels of love (i.e., Intimacy, Passion, and Commitment) between free choice and arranged marriages across five non-Western societies (Igbo from Nigeria, Bhotiya from Himalayas, Meru from Tanzania, Kimeru from Kenya, and Tsimane’ from Bolivia). While our analysis of the combined data from all five cultures did not reveal significant differences in love feelings between free choice and arranged marriages, nuanced variations were observed when each society was analyzed separately. Specifically, spouses from free choice marriages in Bhotiya and Tsimane’ cultures reported higher levels of intimacy than those from arranged marriages. However, in the Bhotiya, this difference was only evident in marriages lasting longer than 10 years. Moreover, spouses from free choice marriages from Bhotiya reported higher levels of passion, while spouses from free choice marriages from Tsimane’ reported higher levels of commitment than individuals from arranged marriages. Conversely, wives from arranged marriages in the Meru culture (Tanzania) exhibited higher intimacy and passion than wives from free choice marriages. There were no significant differences in love scores between free choice and arranged marriages in Kimeru and Igbo.

People raised in Western culture often skeptically view arranged marriages (e.g., Zaidi & Shuraydi, 2002), with the idea of someone else choosing a life partner deemed incompatible with the Western ideal of romantic love. Although our study provides evidence that free choice and arranged marriages might experience romantic love differently, those differences are rather small and, importantly, vary across non-Western and less industrialized cultures. Overall, our data challenges the Western perception that arranged marriages lack love.

Finally, our study offers a new perspective on the kinds of love, as considered from the perspective of the triangular theory of love (Sternberg, 1986). Sternberg acknowledged that romantic relationships might start in some cultures as arranged relationships, with high commitment but low intimacy and passion and named them “empty love.” However, our results suggest that such marriages should not be characterized as lacking love or consisting of only high commitment. Instead, arranged marriages appear to be rich in love, comparably to free choice marriages. Moreover, the general trajectory of love experiences in an average romantic relationship, as hypothesized by the triangular theory of love—with a decline in passion over time and an increase in commitment—might not apply universally in marriages across different non-Western contexts. In the present study, marriage duration was not consistently related (either positively or negatively) to any of the components of love, except in Bhotiya society, where the effects were modest and varied depending on the marriage type (i.e., arranged or free choice). Noteworthy though, differences in love levels with respect to relationship length tend to be subtle (e.g., Sorokowski et al., 2021), and thus, our sample sizes might have been too small to detect them.

Overall, our research partly aligns both with some of the previous studies, which have found that spouses from free choice marriages display more positive attitudes towards their partners and romantic relationships than arranged marriages (Allendorf & Ghimire, 2013; Hortaçsu & Oral, 2010; Lev-Wiesel & Al-Krenawi, 1999; Myers et al., 2005; Pimentel, 2000; Rosenblatt & Cozby, 1972) and also with research, which has not found greater love in free choice marriages compared with arranged marriages (Hoelter et al., 2004; Mir et al., 2016; Shachar, 1991). Importantly, our study is the first to directly examine love components, whereas previous research has often focused on measures such as marital satisfaction (Karandashev, 2015; Xiaohe & Whyte, 1990).

Regarding the trajectory of romantic relationships, our findings contradict Xiaohe and Whyte (1990) study, in which wives with 20–24 years of marital experience from both free choice and arranged marriages reported the highest marital quality. Similarly, our results are at odds with the findings of Karandashev (2015) study, which provided evidence for a constant grow of satisfaction as the years of marriage go by. Some scholars, such as Hudson and Murphy (1980) and Xiaohe and Whyte (1990) have proposed that this may be due to a marriage entering an “empty nest” phase, wherein children leave home and embark on their own lives. This phase could potentially lead to a rekindling of intimacy and satisfaction among long-term couples. However, this does not appear to be the case for the studied societies. The only significant positive link between intimacy and marriage duration was observed in the Tsimane’. Yet, the “empty nest” phenomenon is rather unlikely in this Amazonian population, since Tsimane’ people form “close social ties between extended family” (Ringhofer, 2010, p.78), which would rather prevent the Tsimane’ from feeling social loneliness, associated with the Western concept of “empty nest.”

The observed decline in intimacy over time among individuals from arranged marriages among Bhotiya may be counterintuitive, as partners in such marriages typically lack a shared history (Applbaum, 1995; Chu, 1993). Conversely, establishing a completely new relationship has previously been linked to a gradual growth in intimacy, at least initially (Reis & Patrick, 1996; Wojciszke, 2002). On the other hand, it is worth noting that at the outset of arranged marriages, newlyweds often receive high social support from relatives (Applbaum, 1995; Chowdhry, 2007). This support network may significantly encourage them to exhibit positive and appreciative attitudes towards their spouses (Blood, 1967). More research is needed to examine the potential mechanisms underlying the trajectory of intimate feelings in arranged marriages.

Although the present study is unique in examining love components rather than solely focusing on marital quality in both free choice and arranged marriages, it is not free of limitations. First, the sample size significantly limits the generalizability of the results. While the analysis of the full data revealed no differences in love levels between free choice and arranged marriages (see Table 2), separate analyses within each society indicated some interesting, albeit modest, differences (see Table 3). Larger sample sizes from diverse cultures and regions would contribute to a more comprehensive understanding of these differences in love components between arranged and free choice marriages. Moreover, they could provide further insights into potential sex differences between spouses, which, due to small sample sizes, were not tested in the present research. Second, we treated marriage type as a binary variable, whereas in practice, marriage decision is often influenced by numerous factors and, thus, the type of marriage should rather be characterized on a continuum, with varying degrees of control of the future spouses (Anitha & Gill, 2009). Third, our samples were not representative of their cultures and there were disproportions of participants from free choice and arranged marriages, as well as between women and men—for instance, Meru sample consisted of only women. Moreover, future studies might explore other factors that could explain the differences in love components between free choice and arranged marriages, such as education level, socioeconomic status, and cultural beliefs. Further studies might also focus on explaining the mechanisms underlying the observed cultural differences. Especially insightful might be Trivers’ (1974) parental investment theory or attachment theory (Bowlby, 1982; Mikulincer & Shaver, 2018), which offer fertile ground for formulating new hypotheses (see Diamond, 2004; Scelza et al., 2019). Additionally, it would be interesting to explore the impact of different types of marriages on the long-term stability and relationship satisfaction of spouses.

In summary, our study compared levels of intimacy, passion, and commitment in free choice and arranged marriages across five non-Western societies. The results did not provide evidence for substantial differences between these two types of marriages. However, when each society was analyzed separately, slight variations in love feelings between free choice and arranged marriages across cultures emerged, what requires further investigation.

## Supplementary Information

Below is the link to the electronic supplementary material.

Supplementary file1 (DOCX 11 KB)

## Data Availability

https://osf.io/ztvux/?view_only=3fb2f75c6fa14d6c8d777dffc972b03a.
