# Supplementary material for: Love Components in Free-Choice and Arranged Marriages Among Five Non-Western Populations From Africa, Amazonia, and Himalayas
Source: Arch Sex Behav. 2024 Dec 13;54(1):85–94. doi: 10.1007/s10508-024-03040-y (PMC11782369; doi:10.1007/s10508-024-03040-y)
Supplement: Supplementary file 1 — Supplementary file1 (DOCX 11 KB) [file 10508_2024_3040_MOESM1_ESM.docx]

**Supplementary Material**

**Table S1**

An overview of the cultures (i.e., *Bhotiya*, *Igbo*, *Kimeru*, *Meru*, *Tsimane’*).

| **Culture** | **Country** | **N** | **Women N** | **Free choice N** | **Arranged marriage N** | **Subsistence** | **Population** | **Marriage system** | **Prevalent type of marriage** |
| --- | --- | --- | --- | --- | --- | --- | --- | --- | --- |
| Bhotiya | India | 110 | 55 | 42 | 68 | Semi-nomadic, migratory pastoralists | 27,230 | Monogamy | Usually arranged |
| Igbo | Nigeria | 98 | 47 | 15 | 83 | Agrarian | 34 million | Monogamy (most prevalent, polygamy in the past) | Free choice and arranged |
| Kimeru | Kenya | 124 | 65 | 107 | 17 | Agrarian | 1.3 million | Monogamy (most prevalent, sometimes polygamy) | Usually arranged |
| Meru | Tanzania | 118 | 118 | 21 | 97 | Agrarian | 2 million | Monogamy | Free choice and arranged |
| Tsimane’ | Bolivia | 148 | 75 | 48 | 100 | Farmer-foraging | 8,000 | Monogamy (most prevalent, polygamy in the past) | Arranged but presently also free choice |

**Mean levels of love across cultures**

In Bhotiya, the mean level of love was 2.94 (*SD* = 0.10), in Igbo, the mean level of love was 2.82 (*SD* = 0.23), in Kimeru, the mean level of love was 2.81 (*SD* = 0.25), in Meru, the mean level of love was 2.65 (*SD* = 0.34), in Tsimane’, the mean level of love was 2.64 (*SD* = 0.31).

**Post-hoc analyses**

Detailed Games-Howell post-hoc analyses revealed that there were significant differences in Intimacy scores between *Bhotiya* and *Igbo* (*p* < .001, *d* = 0.31), *Bhotiya* and *Kimeru* (*p* < .001, *d* = 0.34), *Bhotiya* and Tanzanian *Meru* (*p* < .001, *d* = 0.77), *Bhotiya* and *Tsimane’* (*p* < .001, *d* = 0.98), *Igbo* and Tanzanian *Meru* (*p* = .006, *d* = 0.47), *Igbo* and *Tsimane’* (*p* < .001, *d* = 0.67), *Kimeru* and Tanzanian *Meru* (*p* = .007, d = 0.43), and *Kimeru* and *Tsimane*‘ (*p* < .001, *d* = 0.64).

Similarly, there were significant differences in Passion scores between *Bhotiya* and *Igbo* (*p* = .016, *d* = 0.38), *Bhotiya* and Tanzanian *Meru* (*p* < .001, *d* = 0.98), *Bhotiya* and *Tsimane’* (*p* < .001, *d* = 0.93), *Igbo* and Tanzanian *Meru* (*p* < .001, *d* = 0.61), *Igbo* and *Tsimane’* (*p* < .001, *d* = 0.56), *Kimeru* and Tanzanian *Meru* (*p* < .001, d = 0.81), and *Kimeru* and *Tsimane*‘ (*p* < .001, *d* = 0.76).

Finally, there were also significant differences in Commitment scores between *Bhotiya* and *Igbo* (*p* = .016, *d* = 0.42), *Bhotiya* and *Kimeru* (*p* < .001, *d* = 0.74), *Bhotiya* and Tanzanian *Meru* (*p* < .001, *d* = 0.90), *Bhotiya* and *Tsimane’* (*p* < .001, *d* = 0.81), *Igbo* and Tanzanian *Meru* (*p* < .001, *d* = 0.48), and *Igbo* and *Tsimane’* (*p* < .001, *d* = 0.39).

**Shortened and simplified version of Sternberg’s Triangular Love Scale (Sternberg, 1997)**

1. __ is able to count on me in times of need.

2. I am willing to share myself and my possessions with __.

3. I give considerable emotional support to __.

4. I feel that I can really trust __.

5. I share deeply personal information about myself with __.

6. I find myself thinking about __ frequently during the day.

7. I find ___ to be very personally attractive.

8. I would rather be with __ than with anyone else.

9. There is nothing more important to me than my relationship with __.

10. I especially like physical contact with __.

11. I cannot imagine my life without __.

12. I know that I care about __.

13. Because of my commitment to __, I would not let other people come between us.

14. I will always have a strong responsibility for __.

15. I view my relationship with __ as permanent.

16. I plan to continue my relationship with ____________.

17. Even when __ is hard to deal with, I remain committed to our relationship.
